# A data driven policy to minimise the tuberculosis testing cost among healthcare workers

**DOI:** 10.1002/hpm.3496

**Published:** 2022-05-08

**Authors:** Vishnunarayan Girishan Prabhu, Kevin M. Taaffe, Ronald G. Pirrallo, Dotan Shvorin

**Affiliations:** ^1^ Department of Industrial Engineering Clemson University Clemson South Carolina USA; ^2^ Department of Emergency Medicine PRISMA Health ‐Upstate Greenville South Carolina USA

**Keywords:** cost analysis, decision‐making, healthcare policy, mathematical modelling, tuberculosis testing

## Abstract

**Introduction:**

The Centres for Disease Control and Prevention (CDC) mandates that healthcare employees at high‐risk exposure to Tuberculosis (TB) undergo annual testing. Currently, two methods of TB testing are used: a two‐step skin test (TST) or a whole‐blood test (IGRA). Healthcare leadership's test selection must account for not only direct costs such as procedure and resources but also indirect costs, including employee workplace absence.

**Methods:**

A mathematical model based on Upstate South Carolina's largest health system affecting over 18,000 employees on six campuses was developed to investigate the value loss perspective of these testing methods and assist in decision‐making. A process flow map identified the varied direct and indirect costs for each test for four employee types, and 6 travel‐to‐testing‐site times were calculated.

**Results:**

The switching point between testing procedures that minimised total system costs was most influenced by employee salary compared to travel distance. Switching from the current hospital policy to an integrated TST/IGRA testing could reduce TB compliance costs by 28%.

**Conclusions:**

This study recommends an integrated approach as cost‐effective for large health systems with multiple campuses while considering the direct and indirect costs. When accounting for ‘inconvenience costs’ (stress, etc.) associated with visits, IGRAs are recommended irrespective of employee salary.

## Key Points

- High‐risk healthcare workers are required to undergo annual TB testing by CDC
- Two methods of TB testing are used: a two‐step skin test (TST) or a whole‐blood test (IGRA)
- While considering the testing and other travel costs, an integrated testing approach is cost‐effective for large health systems with multiple employee types and campuses.
- However, an IGRA test is recommended for all employees when considering additional inconvenience costs.

## 1 INTRODUCTION

Tuberculosis (TB) is an airborne disease caused by *Mycobacterium tuberculosis*. Although it typically infects the lungs, it can attack other organs, including the spine, brain, and kidney.^1^ TB infections take on one of two forms; latent or active. In the latent form, TB bacteria in the body are suppressed by the immune system. Hence it is asymptomatic and incapable of spreading between individuals. However, infected individuals can progress to active TB disease months to years later.^2^ In the active form, TB is symptomatic and highly contagious. Both forms are detectable by routine TB monitoring, and with properly executed monitoring programs, outbreaks can be mitigated. Healthcare employees are a high‐risk population that is susceptible to TB because of the exposure to unsuspected or undiagnosed TB infected patients.^3^ In 1994, the Centres for Disease Control and Prevention (CDC) published guidelines on infection control policies and practices that are intended to reduce the risk of transmission of TB in healthcare facilities, which were later updated in 2005, 2010, and recently updated in 2019.^4^, ^5^, ^6^ According to the new policy, annual TB testing of healthcare personnel is not recommended unless a known exposure or ongoing transmission at a healthcare facility occurs. However, any existing employees with untreated latent TB infection should receive annual TB testing. Additionally, it is highly recommended to consider using annual TB screening for specific groups at increased occupational risk for TB exposure (e.g., pulmonologists or respiratory therapists) or in particular settings, if a transmission has occurred in the past (e.g., emergency departments). Prior studies have reported that strict maintenance of the CDC policy has helped in reducing the infection of TB among healthcare workers.^3^, ^7^ Clearly, the TB testing policy adopted by a health system to minimise infections affects the programme maintenance cost.

Currently, two widely adopted testing procedures exist; the first and oldest test is the Mantoux tuberculin skin test (TST). This test involves an injection of 0.1 ml of liquid containing 5 tuberculin units into the top layer of the forearm. Once injected, the test is read 48–72 h later by a trained clinician, usually a registered nurse (RN), and the outcome is interpreted using a ruler to measure the diameter of possible induration of area.^8^ If no induration occurs, then the test is reported negative, whereas if the diameter of the indurated area exceeds 10 mm, then the test is considered positive.^9^ In the case of a negative test result, the second step of testing is administered, which is similar to the first step. Whereas, in the case of positive reading, further testing is performed to evaluate for active TB disease, which includes a physical examination and a chest X‐ray. If for any reason, the patient does not return in the stipulated timeframe to have the test read, the testing must be repeated from the first step. Although used predominantly, TSTs are not perfect, and various factors may produce a false‐positive result. Infection with nontuberculous *mycobacterium* is one example. In the case of patients that have previously received the Bacille Calmette‐Guerin (BCG) vaccine, TSTs could record a false‐positive result.^10^ Finally, clinician interpretation of the TST is subjective and requires manual measurement that may influence reporting, especially in borderline cases. Despite being a 2 step, 2‐day process with limitations, TSTs are still adopted by the majority of health systems as the preferred method of testing among employees. This could be attributed to the difference in direct testing material costs and the robust predictive value of TST compared to the variability of IGRA test in longitudinal studies to identify latent tuberculosis bacterial infections.^11^, ^12^, ^16^

The other testing procedure is whole‐blood Interferon‐Gamma Release Assays (IGRAs). IGRAs do not require multiple visits to the facility. Instead of injecting a patient and waiting 48–72 h for an immune response, 1–3 vials of venous blood (total approximately 10 ml) are drawn from the patient and sent to the lab for automated testing. Two IGRAs are available in the US: the QuantiFERON‐TB Gold In‐Tube test (QFT‐GIT) and the T‐SPOT. TB test (T‐Spot). The QFT‐GIT test involves the collection of three tubes of blood (a positive control, a negative control, and a test) that are sent to a laboratory, and they are tested within 16 h of collection.^13^ In the lab, the concentration of interferon‐gamma (IFN‐g) is measured. The T‐Spot test differs from the QFT‐GIT in that only one tube of blood is drawn, and the number of IFN‐g producing cells is measured.^14^ IGRAs have several advantages over the TSTs as they require only a single visit for a blood draw, results can be available in less than 24 h, and prior BCG vaccination does not cause a false‐positive IGRA test result.^10^ However, the IGRAs are costlier than the TSTs. On average, IGRA supply direct costs are $30–38 higher than a TST.^15^ Additionally, blood testing must be completed within a limited time frame, and many facilities do not have the in‐house capability to perform this specific test. Although older studies have reported both increased and decreased sensitivity and specificity analytics for TST compared to IGRAs, more recent studies observed that newly developed IGRAs have higher sensitivity and specificity.^16^, ^17^, ^18^, ^19^, ^20^, ^21^ However, it should be noted that these findings were observed in high‐income countries and cannot be translated broadly. Irrespective of test type, both TST and IGRA tests require the patient's skin to be punctured with a needle and use different testing personnel skill sets.

Under the new CDC policy, the cost incurred by a health system for maintaining TB testing compliance is expected to be reduced as it does not require annual TB testing of all employees. However, it is still essential to conduct a cost analysis for TB screening as it is a time‐consuming process that may lead to a loss of employees' productive working hours. While prior research has focussed on comparing the effectiveness of a TST to IGRA for detecting TB, very few have investigated the overall system costs of these processes.^22^, ^23^ For example, a TST requires a minimum of three (with the potential for five) visits to a facility, compared to a single visit for IGRA testing. Hence it is crucial to identify which TB screening process should be recommended for an employee based on the direct cost and indirect costs of testing.

Previous studies that evaluated the cost of TB testing for a health system have considered only the direct cost associated with the specific testing procedure.^24^ TB testing direct costs are similar to all laboratory testing and include the material cost, procedure cost, and resource cost. Yet, healthcare organisations will incur an additional indirect cost resulting from an employee's absence from the workplace during testing and waiting for results. A detailed view indicates that the indirect cost depends on four factors: time taken to travel to the facility, the time spent for testing, missed employee work hours, and the hourly wage or salary of the employee. A few studies have considered the indirect costs of testing, however to our knowledge, none of the studies have been inclusive of the direct laboratory and indirect personnel costs discussed above.^25^

The simplest cost comparison studies to date have focussed on analysing the cost of maintaining a TST and IGRA separately.^26^ Hence their conclusions were focussed on recommending which specific TB testing method should be adopted by a health system. However, a more nuanced and integrated understanding of how to blend the two testing methods may identify an ideal approach to the cost maintenance of the required TB testing programme. This study was designed to explore whether a switching point exists in the selection of the most economical TB testing method in a large healthcare system.

## 2 METHODS

### 2.1 Mathematical model

This study used the cost and time requirements involved in a test and combined it with two inputs: employee hourly wage and travel time to the facility. A mathematical programing model was developed to identify the switching point for various hourly rates and travel time combinations. This approach was adopted because of its flexibility to investigate different hypotheses, multiple combinations of inputs, and its proven efficacy as a decision‐making tool in healthcare.^27^, ^28^, ^29^ A mathematical programing model includes an objective function (the result of choices or selections made within the model), combined with a set of constraints that represent the feasible or allowable choices based on the assumptions and input parameters provided. This model was derived after the research team interviewed and solicited feedback from hospital policymakers, physicians, and employee health infectious disease stakeholders. Also, policymakers were sensitive to release specific employee hourly pay rates that ranged greatly in this large health care system and recommended general classes of hourly pay. However, to represent the partner health system and include all the employees and their travel distance, we considered various levels of salary and travel distance. These different combinations of the two key input parameters, employee hourly wage and travel time to the facility, were selected and run through the mathematical model. This approach was intended to inform decision‐makers on all of the direct and indirect cost implications of testing decisions. It was hoped that involving healthcare providers, policymakers and stakeholders in the development of the mathematical model would increase confidence in the results and facilitate system adoption. Previous studies have observed that the adoption of new public health policies are limited because of failing to understand the needs of policymakers and end‐users.^30^, ^31^ Specifically, our research team consulted medical directors, physicians, nurses, and Occupational Health and Safety (OHS) managers. Thus, based on the input and recommendations from the policymakers, stakeholders, and end‐users, we were confident to develop a model that provides a recommended testing procedure for a large healthcare system.

### 2.2 Studied population

The study was based on the test costs and policies adopted at PRISMA Health ‐ Upstate, South Carolina, in August 2019. All represented costs are in US dollars. PRISMA Health is the largest not‐for‐profit health organisation in South Carolina, serving more than 1.2 million patients annually with over 18,000 employees. Currently, at PRISMA Health, all incoming employees undergo IGRA QFT‐GIT testing, and approximately 95% of the existing employees who require annual testing undergo a TST.

### 2.3 Study design

After understanding each step and process flow of the 2 TB testing procedures, the mathematical model was created to identify the total cost and time requirements involved to test several employee types and travel distances to the testing facility. The goal of the mathematical decision model was to determine which test, TST or IGRA QFT‐GIT, an employee should undergo in order to minimise the total cost of the system. Although the updated CDC policy does not require annual TB testing for all the healthcare employees, we utilised a modelling approach discussed above that assumes an equal number of employees in wage and location combination.

This study was considered Exempt by the Greenville Memorial Hospital IRB. In this study, although we consider the positive cases under each testing method, we did not differentiate the case of false positives since all employees testing positive would require a compulsory chest X‐ray. Although the false positives were not differentiated based on the probabilities received from the OHS Department, these were included in the positive cases. In general, the positives cases accounted for only 2% for both tests, which reiterates that the false positives would have a minimal effect on our model. Further, we did not consider the case of indeterminate IGRA QFT‐GIT as none of the hospital employees had this case, and prior studies have noted that the likelihood of this condition among adults is less than 0.05%.^32^ The probabilities for each of the scenarios for TST and IGRA QFT‐GIT were obtained from the PRISMA Health OHS Department based on historical testing results.

### 2.4 Skin test (TST) process flow

**FIGURE 1:**
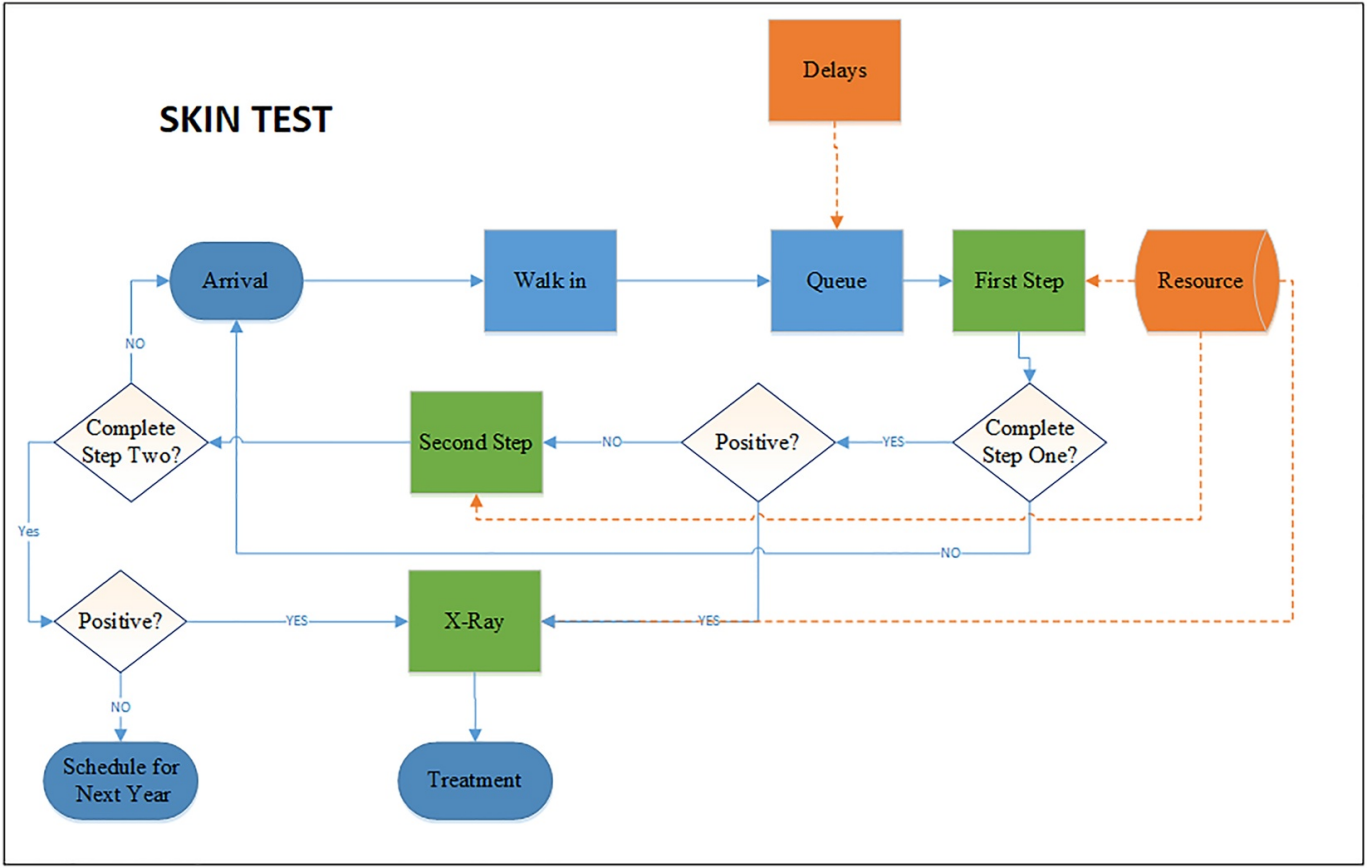
Skin Test (TST) process flow

As mentioned above, the TST testing procedure is a two‐step process and requires multiple visits to the facility. Figure 1 details the process flow of the patient undergoing a TST testing process with different possible scenarios. If an employee misses one of these steps, the testing procedure is repeated from the first step. Thus, six possible scenarios for TST were included in this study.

1. (negative, negative) The employee tested negative in both steps.
2. (negative, positive + chest X‐ray) The employee tested negative in the first step but positive in the second step, followed by a chest X‐ray.
3. (positive + chest X‐ray) The employee tested positive in the first step, followed by a chest X‐ray.
4. (repeat, negative, negative) Same as scenario 1, but the employee does not return within 72 h and must repeat the test from the beginning.
5. (repeat, negative, positive + chest X‐ray) Same as scenario 2, but the employee does not return within 72 h and must repeat the test from the beginning.
6. (repeat, positive + chest X‐ray) Same as scenario 3, but the employee does not return within 72 h and must repeat the test from the beginning.

### 2.5 Blood test (IGRA) process flow

**FIGURE 2:**
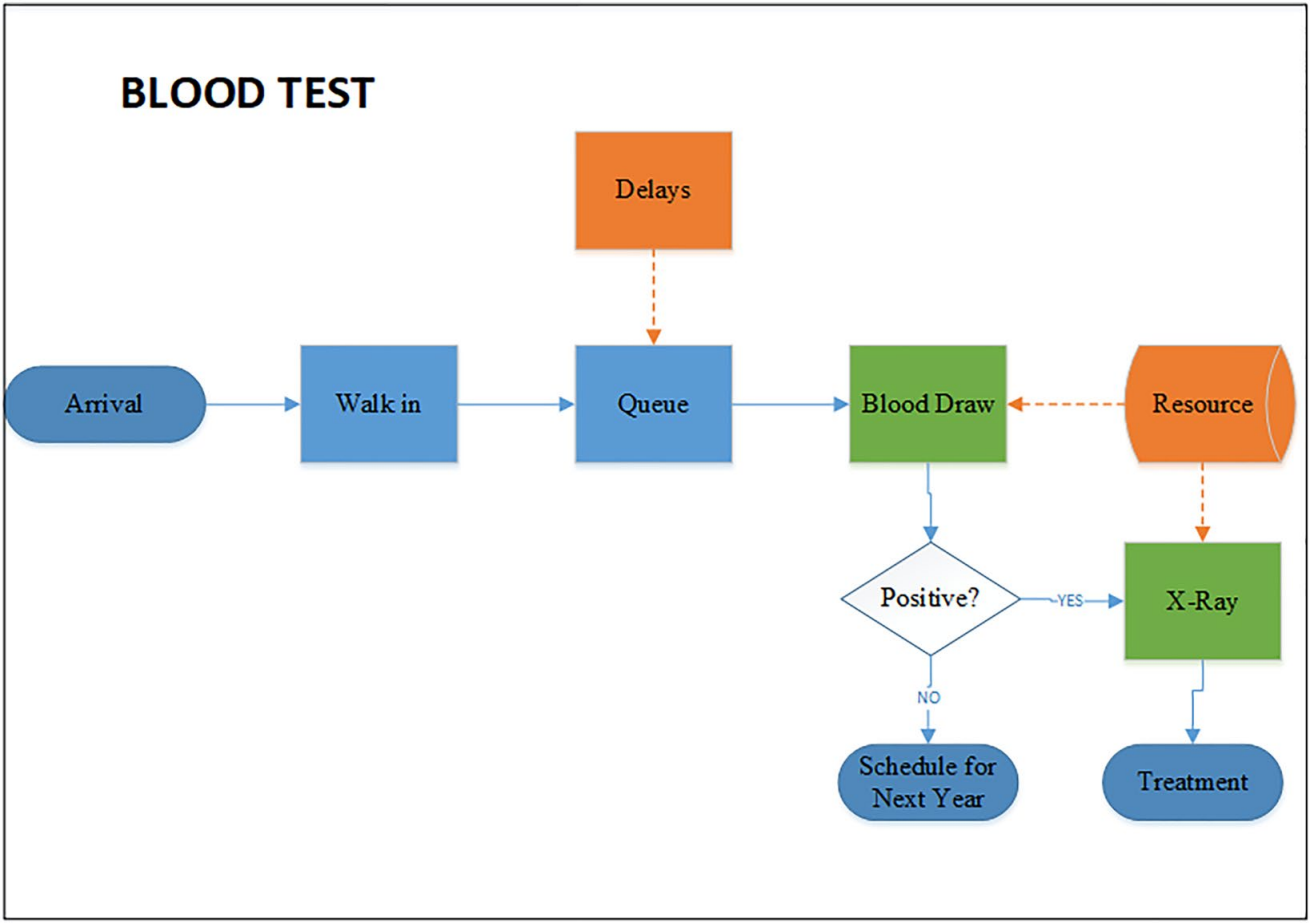
Blood Test (IGRA QFT‐GIT) process flow

The IGRA QFT‐GIT testing procedure is a single‐step testing procedure. Figure 2 details the process flow of the patient undergoing an IGRA testing process with two possible scenarios.

1. (negative) The employee is tested negative for the test.
2. (positive + chest X‐ray) The employee is tested positive and followed by a chest X‐ray.

### 2.6 Notation, data, and model

The model was prepared to capture system costs per pathway based on the type of test, the employee hourly wage, and the employee travel time to/from the facility. The time an employee spends in the lab for testing and waiting in the queue was obtained from the PRISMA Health employee health division, as represented in Table 1. It was assumed that the administration cost of testing was consistent across all tests; the hourly wage of the nurse administering the test was assumed to be $34 based on the South Carolina average nurse salary.^33^ This corresponded to indices of TB test type *j* (where 1 = TST, 2 = IGRA), hourly wage *i*, and travel time *l* in the model. In addition, we denoted *k* as the scenario number for either test type. See formula notation in Table 2.

**TABLE 1:** Testing and waiting times

Process	Time (in mins)
Testing time	3
Wait time for testing	4
Chest X‐ray time	10
Chest X‐ray wait time	7

**TABLE 2:** Model notation for the tuberculosis (TB) test cost equation

Notation	Description
*I*	The set of employee types (or hourly wages)
*J*	The set of TB test options
$K^{j}$	The set of possible scenarios for TB test type *j*
*L*	The set of travel times to/from the facility
$P_{jk}$	Probability of test type *j* in scenario *k*
$S_{i}$	Hourly wage for employee type *i*
$S^{N}$	Hourly wage for the nurse administering the test
$W_{ik}$	Total time spent in the system based on employee type *i* and scenario *k*
$W_{jk}^{N}$	Total time spent in the system by the nurse for test type *j* in scenario *k*
$n_{jk}^{1}$	Number of tests administered for test type *j* in scenario *k*
$n_{jk}^{2}$	0/1 flag indicating whether a chest X‐ray is required for test type *j* in scenario *k*
$n_{ijk}^{3}$	Total number of facility visits based on employee type *i*, test type *j*, and scenario *k*
$T_{l}$	Travel time to/from the facility for distance level *l*
$C_{j}^{1}$	Procedure cost for test type *j*
$C^{2}$	Chest X‐ray cost

### 2.7 Model constraints

Our aim was to develop a general model that could be used in varied hospital systems. Hence, for the analysis, we considered a range of employee types based on their salary (*S*_*i*_), such that it covered the minimum and maximum salary for a full‐time hospital employee. The salaries represented in Table 3 were derived from the wages of the respective employee type in the US.^34^ Similarly, as employee travel times across a large health organisation may vary greatly, the index for travel time (*L*) was divided into 6 10‐min levels, with threshold values ranging from 10 to 60 min, as seen in Table 4. The procedure cost was based on 2019 PRISMA Health costs, and for TST, it was $8 per visit compared to $45 for IGRA QFT‐GIT, and the chest X‐ray cost was $45 under both cases.

**TABLE 3:** Hourly wage for four employee types

Index	Employee‐type *(i)*	Hourly wage (*S*_*i*_) in dollars
1	Physician	150
2	Administrator	48
3	Nurse	30
4	Technician	17

**TABLE 4:** Possible round‐trip travel times between home and facility

Index for travel time (*L*)	Travel time (*T*_*L*_) in mins
1	10
2	20
3	30
4	40
5	50
6	60

Finally, Tables 5 and 6 provide the per‐scenario breakdown of the process flows for TST and IGRA testing. The number of facility visits represents the from/to trips to the testing facility required under each case. In the case of TST, a physician is not required to return for reading the test. Assume that *i* = 1 denotes a physician, then (*n* ^*3*^_*11k*_) shows one less visit than (*n* ^*3*^_*i1k*_), *i ≠ 1*.

**TABLE 5:** Probabilities and number of visits for each scenario under Skin Test (TST)

Scenario (*k*)	Probability (*P*_*1k*_)	# Of tests administered for TST ($n_{1k}^{1})$	# Of facility visits $\left(n_{i1k}^{3}\right)i/1$	# Of facility visits for physicians $\left(n_{11k}^{3}\right)$
1	0.98	2	3	2
2	0.01	2	4	3
3	0.005	1	3	2
4	0.003	3	4	3
5	0.001	3	5	4
6	0.001	2	4	3

**TABLE 6:** Probabilities and number of visits for each scenario under Blood Test (IGRA QFT‐GIT)

Scenario (*k*)	Probability (*P*_*2k*_)	# Of tests administered for IGRA ($n_{2k}^{1})$	# Of facility visits $\left(n_{i2k}^{3}\right)$
1	0.98	1	1
2	0.02	1	2

From the above data, per‐employee cost *Z*_*il*_ for a procedure for each hourly wage *i* and travel time *l* is formulated as:

(1) $$\text{Zil}\;=\underset{j\in J}{\sum }\underset{k\in K^{j}}{\sum }P_{jk}\left(n_{jk}^{1}C_{j}^{1}+S_{i}W_{\text{ijk}}+S^{N}W_{jk}^{N}+n_{jk}^{2}C^{2}+n_{\text{ijk}}^{3}S_{i}T_{l}\right)\;$$

Considering that the overall objective was to identify the switching point from one testing procedure to the other for each (*S*_*i*_, *T*_*l*_) combination such that total system cost is minimised, the restrictions and assumptions do not appear to affect the efficacy of the model.

To execute such an algorithm, and identify the lowest cost selected between the 2 TB test options, the following notation for this optimisation problem is proposed.

Denote *X*_ijl_ as the decision variable such that:

(2) $$X_{\text{ijl}}=\left\{\begin{array}{l} 1,\;\text{if}\;\text{TB}\;\text{test}\;j\;\text{is}\;\text{selected}\;\text{for}\;\text{hourly}\;\text{wage}\;i\;\text{and}\;\text{travel}\;\text{time}\;\text{l,}\\ \text{0,}\;\text{otherwise.}\; \end{array}\right.$$

The resulting cost minimisation problem is shown below.

(3) $$\min Z=\sum_{i\in I}\sum_{j\in J}\sum_{l\in L}X_{\text{ijl}}\left(\sum_{k\in Kj}P_{jk}\left(n_{jk}^{1}C_{j}^{1}+S_{i}W_{ijk}+S^{N}W_{jk}^{N}+n_{jk}^{2}C^{2}+n_{ijk}^{3}S_{i}T_{l}\right)\right)$$

(4) $$\text{subject}\;\text{to}\;\sum_{j\in J}X_{\text{ijl}}=1,\;\forall i\in I,l\in L$$

(5) $$X_{\text{ijl}}\in \left\{0,1\right\},\;\forall i\in I,\;j\in J,\;l\in L$$

This formulation was coded and implemented using Microsoft Excel Solver to identify the switching point between selecting TST testing versus IGRA testing. Although it is hard to quantify the mental stress and other emotional impacts on healthcare workers incurred to complete the testing procedure, the World Health Organisation policy guidelines considers this an essential factor to be addressed.^35^ To account for this, we attached a hypothetical dollar value known as ‘inconvenience cost’ for each visit and incorporated it into the model.

## 3 RESULTS

**FIGURE 3:**
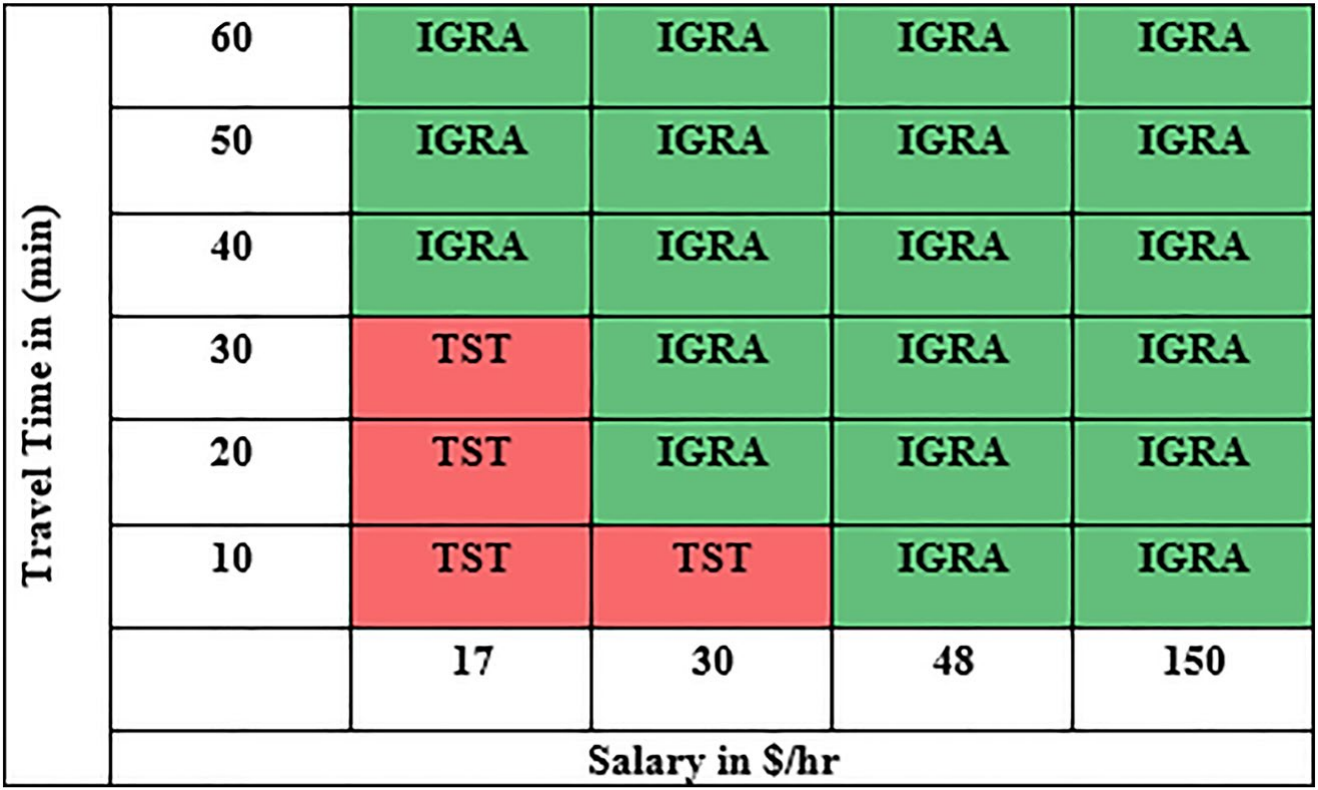
Assignment based on salary and travel time

Using Equation (1), the primary research question, that is, identifying the switching point between selecting TST testing versus IGRA testing across a set of hourly wage and travel time combinations, was achieved. Figure 3 below represents the switching point from one testing procedure to the other based on hourly wages and travel time.

**FIGURE 4:**
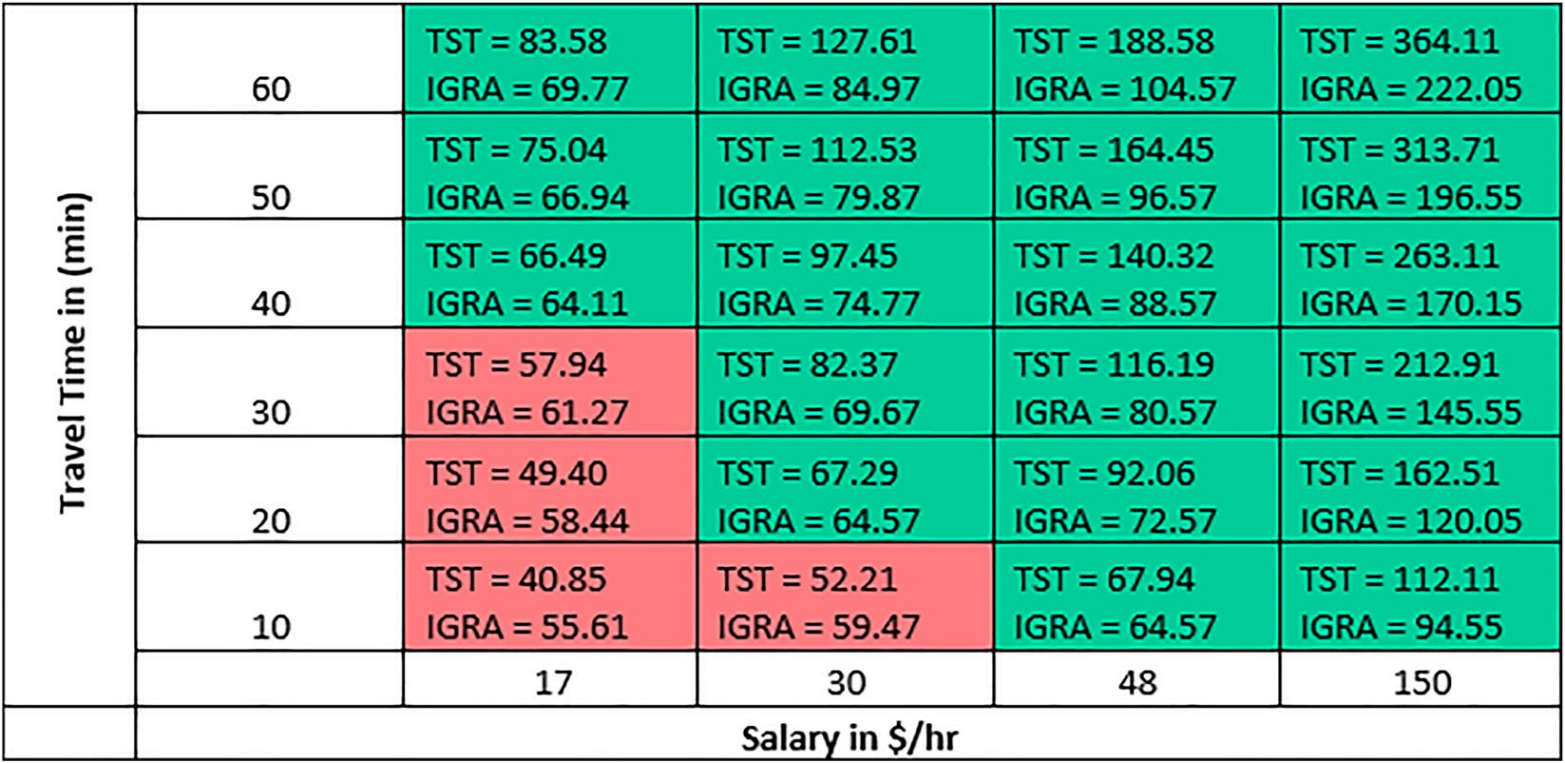
Individual test cost for each combination of salary and travel time

Equation (3) represents the objective function to be minimised to obtain a minimum TB testing cost for the healthcare system. As mentioned earlier, currently, 95% of the existing PRISMA Health employees who require annual testing undergo a TST. The total cost *Z* under the current PRISMA Health policy to the new blended policy derived from the model was compared assuming 100 employees within each hourly wage *i* and travel time *l* combination, for a total of 2400 employees. These numbers were selected to demonstrate to the policymakers the influence of each variable. Using an equal number of employees in each hourly wage *i* and travel time *l* combination made it easier for policymakers to see the switching point. In addition, ad hoc changing the number of employees in each group further reinforced the influence of the indirect costs to policymakers with a better understanding of the individual cost of the testing policies under each scenario. Figure 4, which represents the cost for an employee under each combination. Finally, we represent the total cost for the current and new blended policy in Table 7.

**TABLE 7:** Total cost (*Z*) under two policies

**Policy**	**Total Cost**
Total cost (*Z*) under the current PRISMA health policy	$311,108
Total cost (*Z*) under a new blended policy	$223,235

When we incorporate an inconvenience cost per visit, as mentioned earlier, even a $10 cost per visit would lead to recommending an IGRA irrespective of hourly salary. It is interesting that such a small cost value significantly impacts the decision‐making as TSTs require more visits compared to blood tests. The significance of even a small ‘inconvenience cost’ may be unrecognised by decision makers.

## 4 DISCUSSION

Mathematical modelling can assist healthcare system decision‐makers in understanding the implications of employee TB compliance testing programs. This model distills the known direct costs of Mantoux two‐step tuberculin skin testing compared to Interferon‐Gamma Release Assay laboratory testing and value loss perspectives of employee time into a definable switching point. Regardless of direct cost variations, a switching point between testing procedures that minimised total system costs was most influenced by employee salary. Based on the model, an employee who is paid more than $48/hour should undergo IGRA QFT‐GIT blood testing irrespective of the travel time. As the employee pay rate decreases to $30/hour, TST testing becomes more economical if travel times are less than 10 min. Although actual costs and potential dollars saved depends on TB testing compliance rules and regulation, it appears that a blended model of TB testing may be the most cost‐effective approach for a large healthcare employer with multiple campuses.

Clearly, just looking at the cost to purchase a TB test (TST or IGRA QFT‐GIT) is suboptimal from both an employee and employer point‐of‐view. Multiple factors must be considered when adopting a system‐wide policy for TB testing. Employees and decision‐makers may have different value metrics. For example, employees may value the ease of a single visit with no requirement for follow‐up, and the employer may value the reduced costs of one testing procedure over the other. Although cost‐effective, the proposed blended TB testing model with a switch point may have the challenge of messaging to employees that avoid perceptions of bias and discrimination of lower‐paid employees for successful implementation. Emotional or mental stress on employees requiring multiple visits may also be considered. To test this, we considered an added scenario by incorporating an additional cost defined as the ‘inconvenience cost’ and observed that even a cost as low as $10 significantly impacts the model. This suggests all providers be recommended for an IGRA test. Thus, decision makers should be cautioned not to overlook or minimise this important variable when creating policy.

Although this mathematical model does not address all the complex factors that decision‐makers must address, computing the individual cost of both tests with readily available and understood inputs allows the policymaker to see the system impact of their decision. For example, a smaller health system with limited testing facilities and great travel distances might opt for every employee to get an IGRA test as it saves the time of multiple visits for a minimal increase in cost. Using this mathematical model, large healthcare systems may better understand the influence of multiple factors, including a budget, testing constraints, employee wage, and lost work has on their decisions. Additional host factors such as immunosuppression and known high risk population care givers are not necessarily addressed in the model's output; yet they are critical in ensuring appropriate employee TB testing.

With the current level of focus on healthcare cost reduction, decision‐makers understand the importance of objective data. Mathematical modelling, even with its limitations, can assist decision‐makers in assessing the ramifications of their decisions prior to implementation. One cost reduction extreme approach would have staff complete their TB testing on personal time, thus reducing employee work lost to zero. The regulatory, ethical and employee morale and job satisfaction issues would likely be non‐practical. The caveat of this research is that complete dependence on mathematical modelling may lead to unrealistic solutions. Thus, importance of decision‐makers understanding of the inputs into a mathematical model cannot be understated.

## 5 CONCLUSION

This research focussed on minimising the TB testing cost among healthcare workers by considering both the direct and indirect costs associated with two of the most commonly used testing procedures. It appears that mathematical modelling can inform decision‐makers on the total costs of an employee TB testing policy for a large healthcare system. The study observed that employee hourly salary and travel time to testing facilities are most influential in identifying a switching point in a blended TST and IGRA QFT‐GIT TB testing compliance programme that minimises system costs. Yet when incorporating an employee ‘inconvenience cost’ for each visit, we observed IGRAs to be more affordable for all providers. Researchers should include emotional stress, mental stress, and other behavioural factors in a model and may be surprised by their influence on the final costs.

## CONFLICT OF INTEREST

The authors have no conflicts of interest to declare.

## ETHICS STATEMENT

Not applicable.

## Data Availability

The data that support the findings of this study are openly available in the tables in the paper.
